# The hypothesis that Helicobacter pylori predisposes to Alzheimer’s disease is biologically plausible

**DOI:** 10.1038/s41598-017-07532-x

**Published:** 2017-08-10

**Authors:** Felice Contaldi, Federico Capuano, Andrea Fulgione, Riccardo Aiese Cigliano, Walter Sanseverino, Domenico Iannelli, Chiara Medaglia, Rosanna Capparelli

**Affiliations:** 1 Department of Agriculture, University of Naples “Federico II”, Portici, 80055 Italy; 2 Department of Food Microbiology, Istituto Zooprofilattico Sperimentale del Mezzogiorno, Portici, 80055 Italy; 3 Sequentia Biotech, Edifici CRAG, Campus UAB, Bellaterra (Cerdanyola del Vallès), Barcelona, 08193 Spain; 4 Department of Immunology, Weizmann Institute of Science, Rehovot, 76100 Israel

## Abstract

There is epidemiological evidence that *H. pylori* might predispose to Alzheimer’s disease. To understand the cellular processes potentially linking such unrelated events, we incubated the human gastric cells MNK-28 with the *H. pylori* peptide Hp(2-20). We then monitored the activated genes by global gene expression. The peptide modulated 77 genes, of which 65 are listed in the AlzBase database and include the hallmarks of Alzheimer’s disease: APP, APOE, PSEN1, and PSEN2. A large fraction of modulated genes (30 out of 77) belong to the inflammation pathway. Remarkably, the pathways dis-regulated in Alzheimer’s and Leasch-Nyhan diseases result dis-regulated also in this study. The unsuspected links between such different diseases – though still awaiting formal validation – suggest new directions for the study of neurological diseases.

## Introduction

Alzheimer disease (AD) is a progressive, age-influenced neurodegenerative disease. AD can display an early or late onset depending upon the genome, diet and lifestyle of the patient^1,2^. The hallmark of both these forms of AD is the presence of neurofibrillary tangles (NFTs) of the phosphorylated protein tau and insoluble fibrils and plaques of the amyloid-β peptide (Aβ_42_)^3^. Early onset AD (EOAD) is a rare form of AD with a prevalence of 5.3 × 10^5^ people at risk^4^. About 85% of the patients affected by EOAD display rare mutations in the amyloid precursor protein (*APP*) or the presenilin (*PSEN1, PSEN2*) loci^4^. Copy number variants (CNVs) have been detected in 21 unrelated EOAD patients with no mutations at the main *APP* or *PSEN* loci^4^. The more frequent late onset form of AD (LOAD) is associated with mutations of the apolipoprotein E (*APOE*) gene. The *APOE-ε4* allele displays dosage effect: the proportion of affected subjects is 47% for heterozygotes (2/4 or 3/4) and 91% for homozygotes (4/4)^5^. The *APOE*-ε4 allele is a risk factor also for EOAD^6^. More recent studies have described 19 genes (11 of which are new) associated (P < 5 × 10^−8^) with LOAD^7^.

*Helicobacter pylori* (*H. pylori*) infection is limited to the human stomach^8^. This Gram-negative bacterium causes gastritis, peptic ulcer and more rarely gastric cancer. The life-time risks of developing ulcers or gastric carcinoma are 10–20% and < 1%, respectively^8,9^. *H. pylori* infection is also associated with non-gastric diseases: AD, Parkinson’s disease, atherosclerosis, and cardiovascular ischemia^10–12^. In the case of AD, two genetic association studies – both carried out on small numbers of patients of European ancestry – report an association between AD and *H. pylori* infection^13,14^. However, a larger study – carried out on Japanese patients - did not confirm the association^15^.

Recently, two of our patients with *H. pylori* infection^16^ manifested symptoms of AD. This observation stimulated the present study, aimed at detecting a potential biological link between *H. pylori* infection and AD. Case-control studies suffer from low replication^17,18^, resulting from confounding factors such as genetic heterogeneity^19^, pleiotropy^20^, population stratification^21^, or epistasis^22^. To test our hypothesis, we therefore opted to use the RNA sequencing technology (RNA-seq) that has become particularly attractive for gene expression studies because highly reproducible^23^. In addition, being independent of assumptions about the genes involved, RNA-seq can lead to the identification of new gene products or pathways.

The Hp(2-20) peptide - derived from the *H. pylori* ribosomal protein L1^24^ - is a ligand of the formyl peptide receptors (FPRs) FPR1, FPRL1, and FPRL2^25^. FPRs are seven transmembrane G protein-coupled receptors which regulate inflammation, a critical player in AD^26^. The gastric mucosal cell line MKN-28 expresses both the FPRL1 and FPRL2 proteins^25^. The Aβ_42_ peptide - the dominant component of amyloid plaques found in the brains of AD patients^3^ – is also a ligand for FPRL1^27^. Strikingly, synthetic and secreted humanin peptides protect neural cells by inhibiting the access of Aβ_42_ to FPRL1^28^. The FPRL1 ligands - in addition to Aβ_42_ and humanin- also include the host-derived agonists annexin A1 and lipoxin A4^27^, which display strong anti-inflammatory activity and promote apoptosis and phagocytosis at the site of inflammation^27^. Notably, FPRL1 displays copy number variants associated with extreme forms of AD^4^.

Hp(2-20) upregulates the VEGF-A pathway expression at the mRNA and protein levels^25^ and activates the ERK and Akt pathways that in turn cooperate with the VEGF-A pathway^29^. VEGF-A plays a crucial role in mitigating neural injury and promoting neurogenesis and brain repair in AD patients^29^. The astrocytes from AD patients display increased VEGF-A immunoreactivity, which is interpreted as a compensatory mechanism countering the reduced vascularity occurring in AD patients^30^. At the same time, single nucleotide polymorphisms of the *VEGF-A* promoter that predispose to AD are also known^31^. Cumulatively, these traits make the MKN-28 cells and the Hp(2-20) peptide both well suited for tracing a potential thread connecting *H. pylori* infection with AD. Thus, MKN-28 cells were incubated with the peptide Hp(2-20) and the differentially expressed genes analyzed for known transcriptional associations with AD. The Hp(2-20) peptide induced the transcription of 5911 genes, of which 77 are listed in the AlzBase database.

## Results

### Identification of genes with altered expression levels

To understand the cellular processes potentially linking *H. pylori* infection and AD, we incubated the human gastric cell line MKN-28 with the peptide Hp(2-20) alone (condition A), the *H. pylori* growth broth alone (condition B), or with both the peptide and the growth broth (condition C). We then monitored the genes activated under these conditions by RNA-seq.

**Figure 1:**
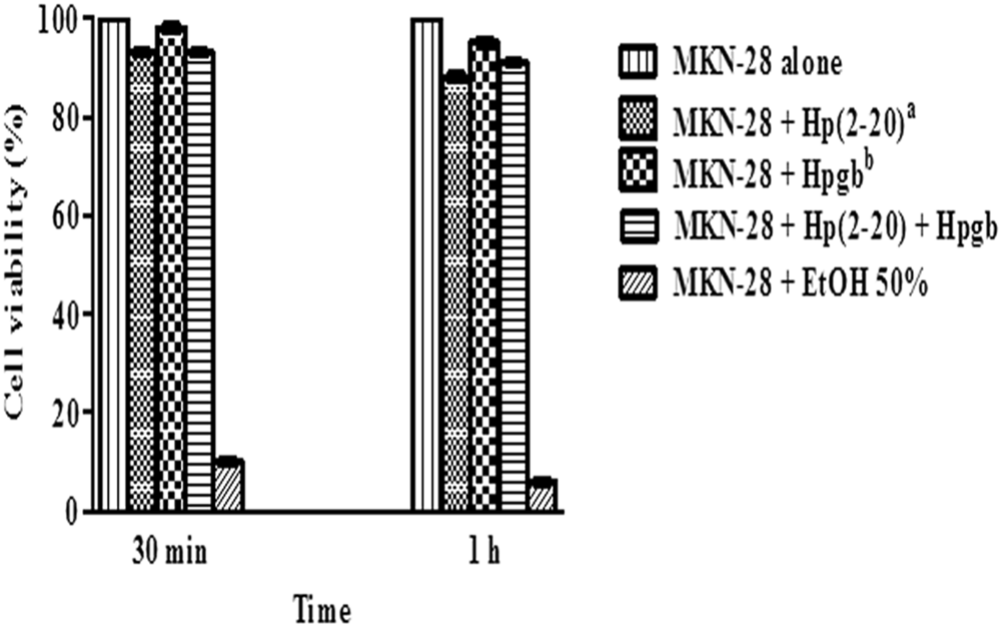
Analysis of MKN-28 cells viability. Results are representative of three independent experiments. Each value is the mean ± SD of three replicas. Statistical analysis was carried out with the GraphPad Prism version 5.03 (GraphPad, La Jolla, CA, USA). Cell viability was statistically significant (*P* < 0.001) in each case; ^a^Hp(2-20) (2 × 10^–5^ M); ^b^Hpgb = *H. pylori* growth broth (140 µl/well).

**Figure 2:**
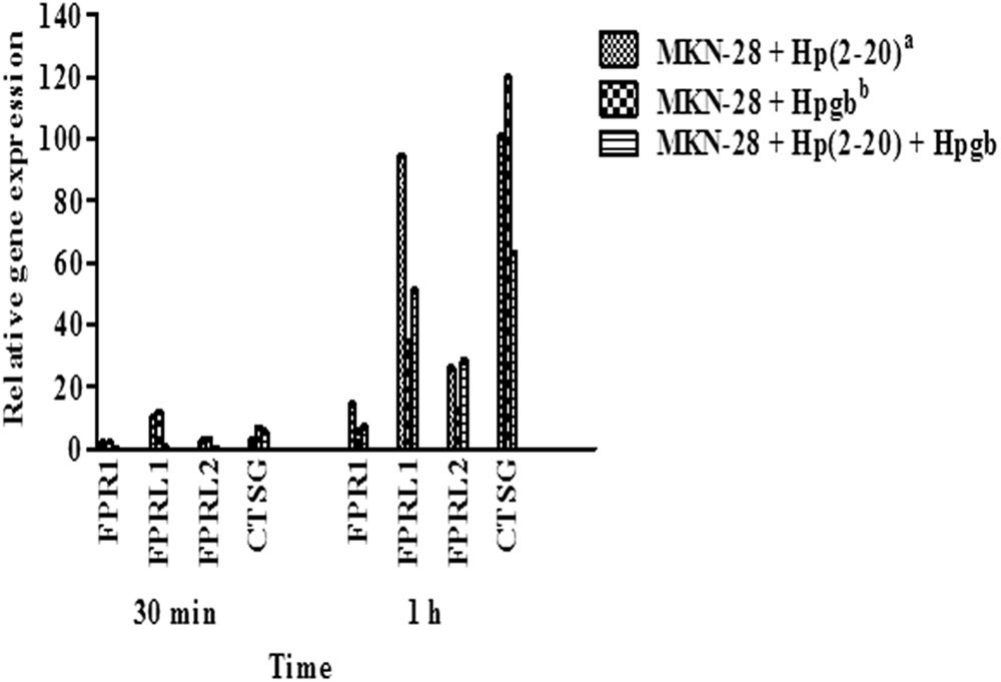
MKN-28 mRNAs levels of FPR1, FPRL1, FPRL2 and CTSG. Results are representative of three independent experiments. Each value is the mean ± SD of three replicas. Expression values were normalized against the human glyceraldehydes-3-phosphate dehydrogenase (GAPDH) gene. Stability assay, carried out using the BestKeeper tool, indicated that GAPDH was more stable then ACT-β at 30 (1.03 vs 2.89) and 60 min (1.89 vs 3.29). Statistical analysis was carried out with the GraphPad Prism version 5.03 (GraphPad, La Jolla, CA, USA). Differences in expression levels between 30 min and 1 h are all significantly different (P < 0.001); ^a^Hp(2-20) concentration was 2 × 10^−5^ M; ^b^Hpgb = *H. pylori* growth broth (140 µl/well).

First, we performed a time course experiment to determine the optimal exposure time of MKN-28 cells to the conditions A, B, and C (Fig. 1). This preliminary pilot test was limited to some genes with a role in AD: *FPR1*, *FPRL1, FPRL2*, and *CTSG*. The latter codes for the cathepsin G, a protease cleaving the Aβ_42_ peptide from the APP precursor protein^32^ and a ligand for FPR1^33^. From these experiments, we concluded that 1 h incubation time of MKN-28 cells is the optimal: it induces the expression of AD-related genes, mimicking this disorder at the cellular level without being cytotoxic (Fig. 2).

**Figure 3:**
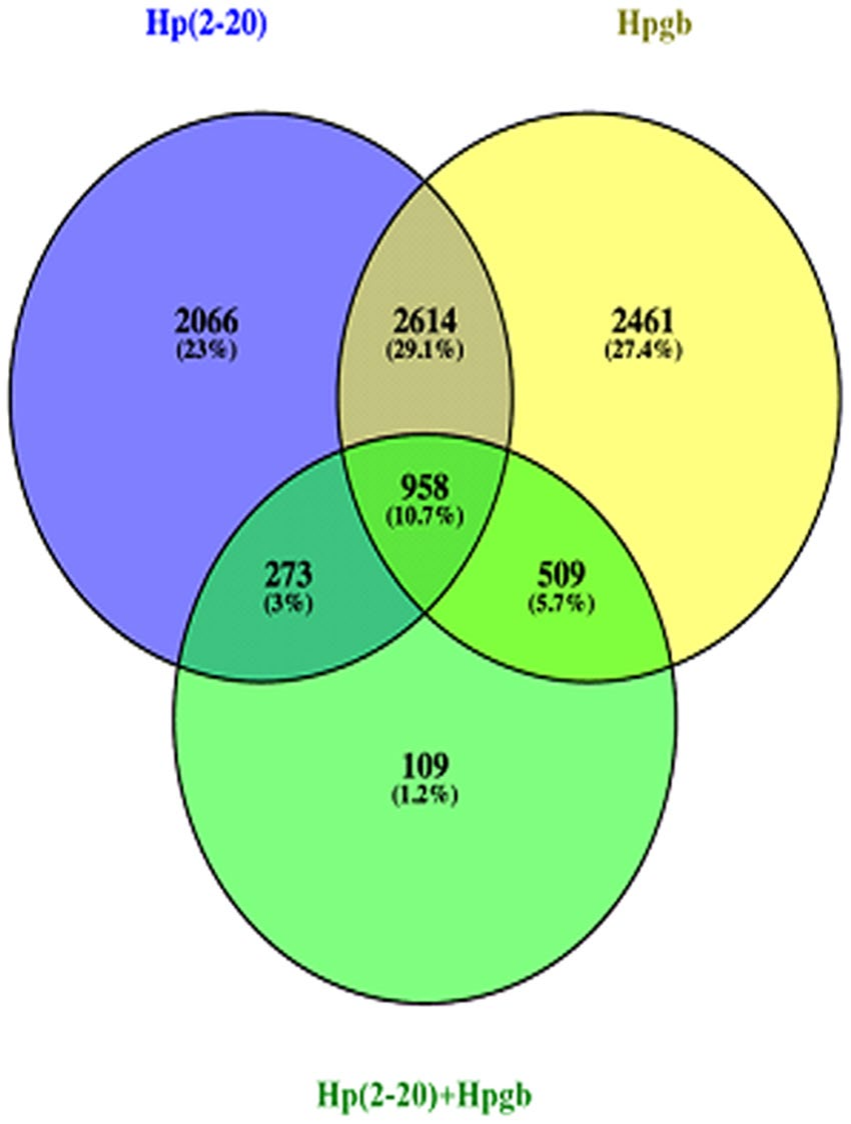
Venn diagram presentation of unique and common genes dysregulates in the presence of Hp(2-20) (**A**), the *H. pylori* growth broth (**B**), or both (**C**).

Next, using RNA-seq, we measured the changes in gene expression of MKN-28 cells upon 1 h exposure to conditions A, B, and C. We identified 958 genes whose expression was affected by all the tested conditions. Hereafter we refer to these genes as “common”, while we name “unique” those whose expression changed exclusively upon exposure to one of the conditions. Specifically, we found 2066 genes unique to condition A, 2641 unique to condition B and 109 unique to condition C (Fig. 3). RNA-seq analysis was carried out on three biological replicates for each condition. Controls were untreated MKN-28 cells. Differential expression across the A, B, and C conditions, involved analysis of an average of 30 million reads for each sample; 90% of them mapped uniquely on the human reference genome. The total number of genes differentially expressed in conditions A, B and C were about 5900, 6500 and 1800, respectively (Fig. 3).

### Common genes with altered gene expression

**Figure 4:**
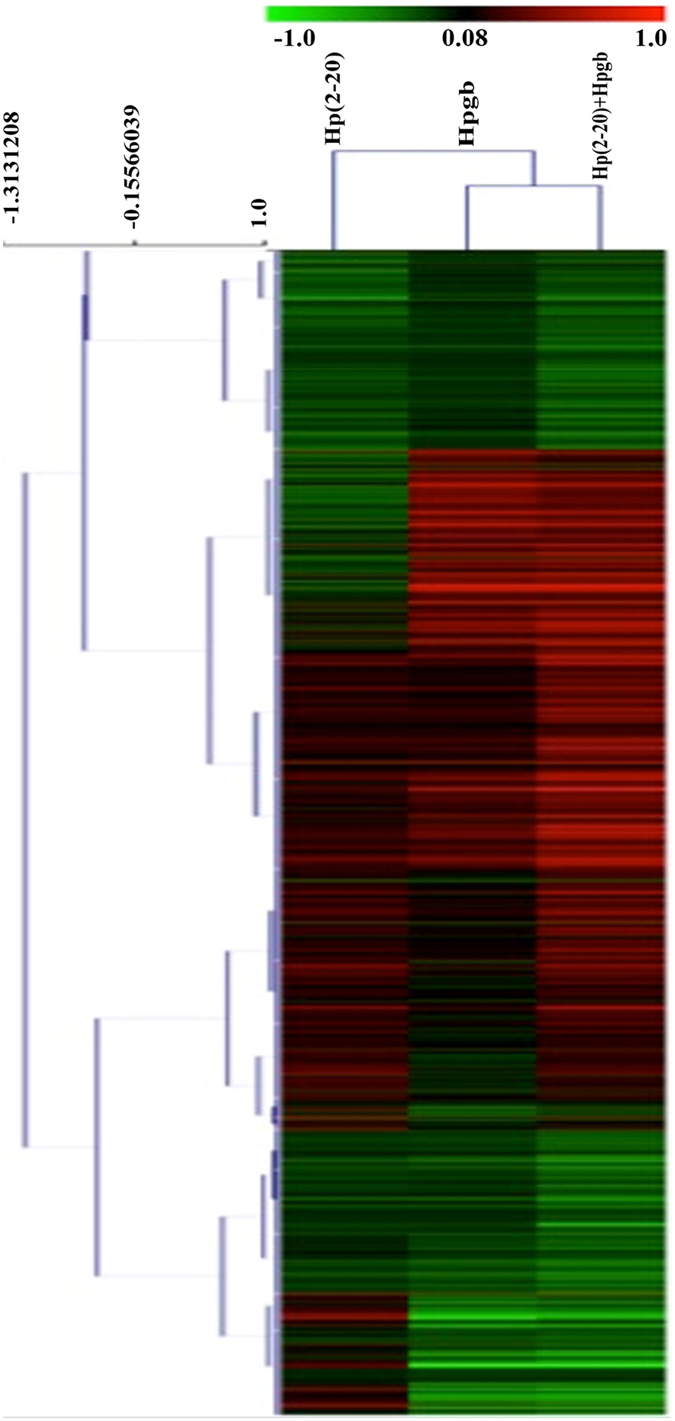
Heat map of the 958 common genes. Columns represent differences in expression levels (from green (down-regulated) to red (upregulated) in the presence of Hp(2-20) (**A**), the *H. pylori* growth broth (**B**), or both (**C**). Heat map and hierarchical clustering were obtained based on log2 fold-change.

**Figure 5:**
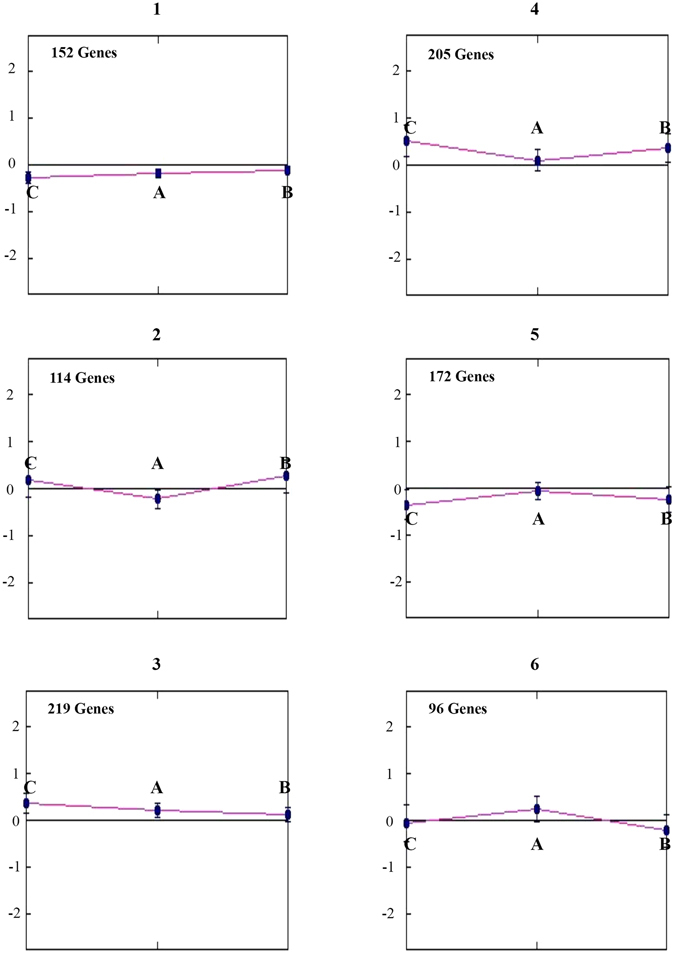
Classification of 958 common genes according to the QT-CLUST tool. Genes displaying similar trends in at least two conditions are clustered together. The letters A, B, and C indicate gene expressions in the presence of Hp(2-20), the *H. pylori* growth broth, or both, respectively.

We first analyzed the common genes with MultiExperiment viewer (MeV) and the QT CLUST tools. The former displays single gene expressions under the three conditions (Fig. 4). The latter divides genes into six clusters on the basis of their similar trends in at least two conditions. In particular, the genes of clusters 3 and 4 are upregulated across conditions A, B, and C, while the genes of clusters 1 and 5 instead are downregulated across the same conditions. The genes of cluster 2 are downregulated in A and upregulated in B and C. On the contrary, the genes of cluster 6 are upregulated in A and downregulated in B and C (Fig. 5). The gene ontologies associated with each cluster are reported in Supplementary Table S1.

Inspection of Fig. 5 shows that gene modulation induced by the Hp(2-20) peptide is clearly different when used alone (1, 3, 4, 5) or in combination with *H. pylori* growth broth (2, 6). On practical grounds, the above results clearly indicate that the concurrent use of two preconditioning factors might interfere with the correct understanding of single genes expression.

### Unique genes displaying altered expression in the presence of the *H. pylori* growth broth

**Figure 6:**
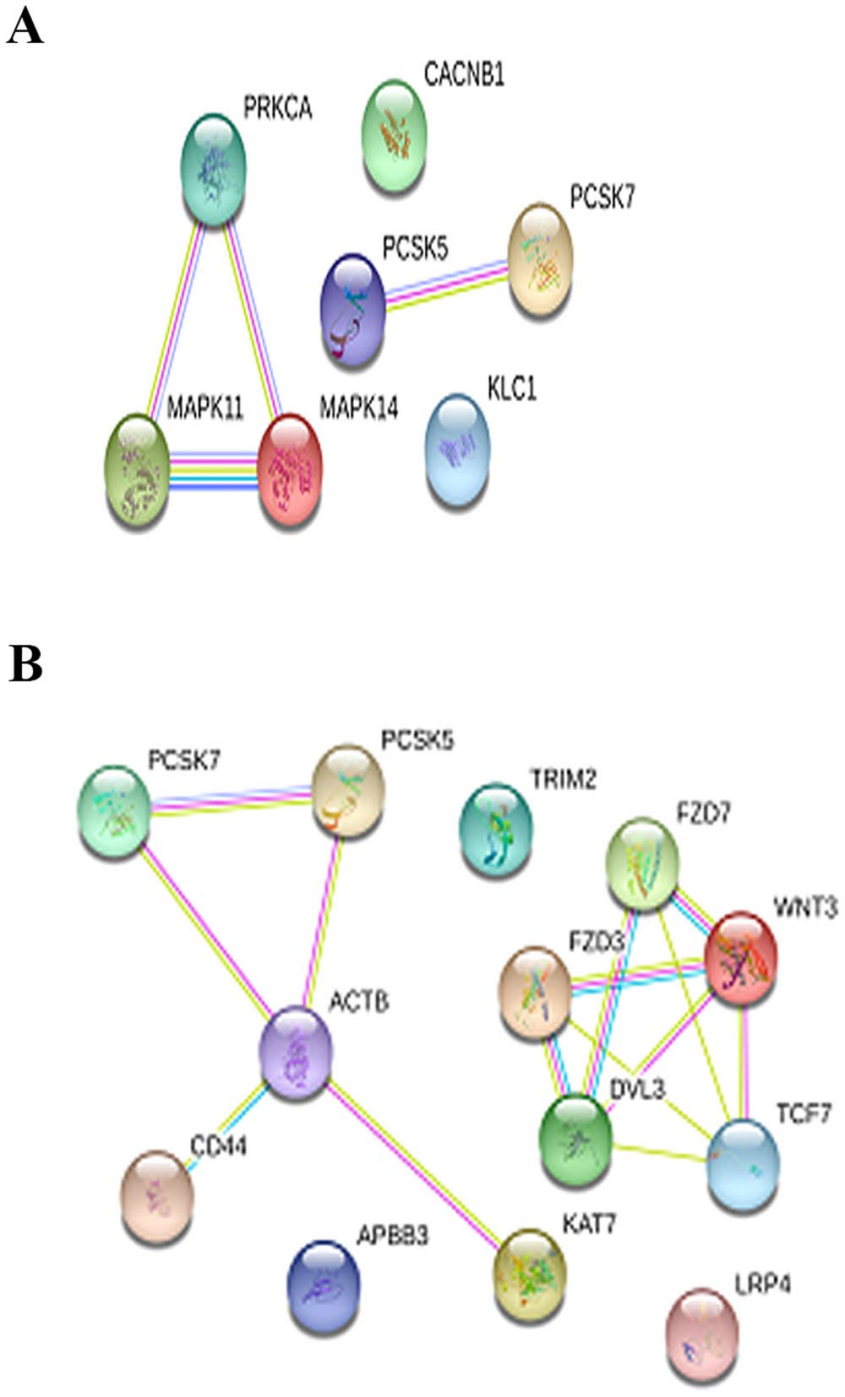
Network analysis of Alzheimer disease-amyloid secretase (**A**) and of Alzheimer-disease presenilin (**B**) genes catalogued in the Panther database and activated by the *H. pylori* growth broth. Lines indicate interactions between proteins (nodes).

When the preconditioning factor was the *H. pylori* growth broth, the majority of the genes with modulated expression were associated with *H. pylori* infection (data not shown). The relatively few genes associated with AD represented either the Alzheimer’s disease-amyloid or the Alzheimer-disease presenilin pathways. However, these genes displayed limited interaction (Fig. 6). We then analyzed the data from cells preconditioned with Hp(2-20) alone.

### Genes displaying altered expression in the presence of the Hp2-20 peptide

We identified 2066 genes which, following preconditioning with Hp(2-20), displayed a change in expression levels (data not shown). The list includes genes from 131 signaling pathways (Supplementary Table S2), some of which are indirectly relevant to AD (inflammation, angiogenesis, VEGF and Wnt signaling) (Table 1) and some more directly relevant (Alzheimer disease-amyloid secretase, and Alzheimer disease-presenilin) (Table 2). These pathways include 77 genes among which are the following hallmarks of AD: *WNT10B, DKK1*, and *FZD5* (up-regulated); *TCF7L2* and *LRP6* (down-regulated); *ANXA1, PSEN-1, PSEN-2, APOE, CTNNB1* (up-regulated); *MTRNR2L2* (down-regulated).

**Table 1:** Pathways represented among the 77 genes associated with AD and differentially expressed upon activation with Hp(2-20).

Pathways	Genes
Alzheimer disease-amyloid secretase pathway (P00003)	*PRKCI, MAPK8, MAPK13, ADAM9, MAPK3, ADAM17, PRKCQ, PKN2, APP, PSEN1, PSEN2, PRKACA*
Alzheimer disease-presenilin pathway (P00004)	*RBPJ, ACTL8, APBB2, FZD5, WNT10B, LRP6, ADAM17, NECTIN1, TCF7L2, PSEN1, PSEN2, NOTCH1, CTNNB1, CTNNA1, APP*
Angiogenesis (P00005)	*PRKCI, FRS2, RBPJ, RASA1, FZD5, MAPK8, PIK3CB, NRAS, WNT10B, SOS2, PRR5, PLA2G4A, MAP3K1, MAPK3, MAP2K4, SRC, PTPN, PLD2, PLCG2, RHOC, BRAF, TCF7L2, PRKCQ, PRKACA, NOTCH1, CTNNB1*
VEGF signaling pathway (P00056)	*PRKCI, PIK3CB, NRAS, PRR5, PLA2G4A, MAPK3, PLCG2, BRAF, PRKCQ, PRKACA*
EGF receptor signaling pathway (P00018)	*PPP4C, PRKCI, PPP2CB, RASA1, RHOG, YWHAH, MAPK8, PIK3CB, NRAS, GAB1, SOS2, SPRY4, NEK1, MAPK13, MAP3K1, PPP6, MAPK3, MAP2K4, RASA2, PLCG2, BRAF, PRKCQ, CBLB, ERBB3, PRKACA*
Wnt signaling pathway (P00057)	*LRP6, PRKCI, PPP2CB, PRKACA, PRKCQ, ARRB1, ACTL8, GNAQ, FZD5, TCF7L2, WNT10B, CTNNB1, CTNNA1*
Inflammation mediated by chemokine and cytokine signaling pathway (P00031)	*ARRB1, PRKACA, ACTL8, RHOG, GNAQ, REL, PIK3CB, NRAS, IFNAR1, NFKB1, ARPC1B, PDPK1, IKBKE, PLA2G4A, ROCK1, PRKX, GRK6, MAPK3, ARPC4, PLCG2, RELA, SOCS6, RHOC, BRAF, PTEN, JUNB, FPR1, FPR2, FPR3, ANXA1*

*The FDR value of listed genes was <0.05.

**Table 2:** Genes of Alzheimer disease-amyloid secretase and Alzheimer disease-presenilin pathways catalogued in the Panther database and differently expressed upon activation with Hp(2-20).

Pathways name	Gene ID	Gene name	FDR	log2FC
*Alzheimer disease-amyloid secretase pathway*	*PRKCI*	Protein kinase C iota type	3.11E-02	−0.11
	*MAPK8*	Mitogen-activated protein kinase 8	4.24E-02	−0.15
	*MAPK13*	Mitogen-activated protein kinase 13	3.51E-02	0.10
	*ADAM9*	Disintegrin and metalloproteinase domain-containing protein 9	2.51E-02	−0.08
	*MAPK3*	Mitogen-activated protein kinase 3	1.52E-02	0.23
	*ADAM17*	Disintegrin and metalloproteinase domain-containing protein 17	2.55E-02	−0.26
	*PRKCQ*	Protein kinase C theta type	3.03E-02	−0.22
	*PKN2*	Serine/threonine-protein kinase N2	1.73E-02	−0.26
	*APP*	Amyloid Beta Precursor Protein	2.94E-02	0.04
	*PSEN1*	Presenilin 1	2.77E-02	0.35
	*PSEN2*	Presenilin 2	1.83E-02	0.49
	*PRKACA*	Protein Kinase CAMP-Activated Catalytic Subunit Alpha	3.50E-02	0.14
*Alzheimer disease-presenilin pathway*	*RBPJ*	Recombining binding protein suppressor of hairless	2.48E-02	−0.21
	*ACTL8*	Actin-like protein 8	3.14E-02	−0.48
	*APBB2*	Amyloid beta A4 precursor protein-binding family B member 2	2.99E-02	−0.18
	*FZD5*	Frizzled-5	4.99E-02	0.10
	*WNT10B*	Protein Wnt-10b	2.72E-02	0.30
	*LRP6*	Low-density lipoprotein receptor-related protein 6	2.48E-02	−0.22
	*ADAM17*	Disintegrin and metalloproteinase domain-containing protein 17	2.55E-02	−0.26
	*NECTIN1*	Nectin-1	3.86E-02	0.10
	*PSEN1*	Presenilin 1	2.77E-02	0.35
	*PSEN2*	Presenilin 2	1.83E-02	0.49
	*NOTCH1*	Notch 1	1.21E-002	0.36
	*CTNNB1*	Catenin Beta 1	8.98E-03	−0.18
	*CTNNA1*	Catenin Alpha 1	1.87E-02	0.30
	*APP*	Amyloid Beta Precursor Protein	2.94E-02	0.04
	*TCF7L2*	Transcription factor 7-like 2	3.14E-02	−0.18

**Figure 7:**
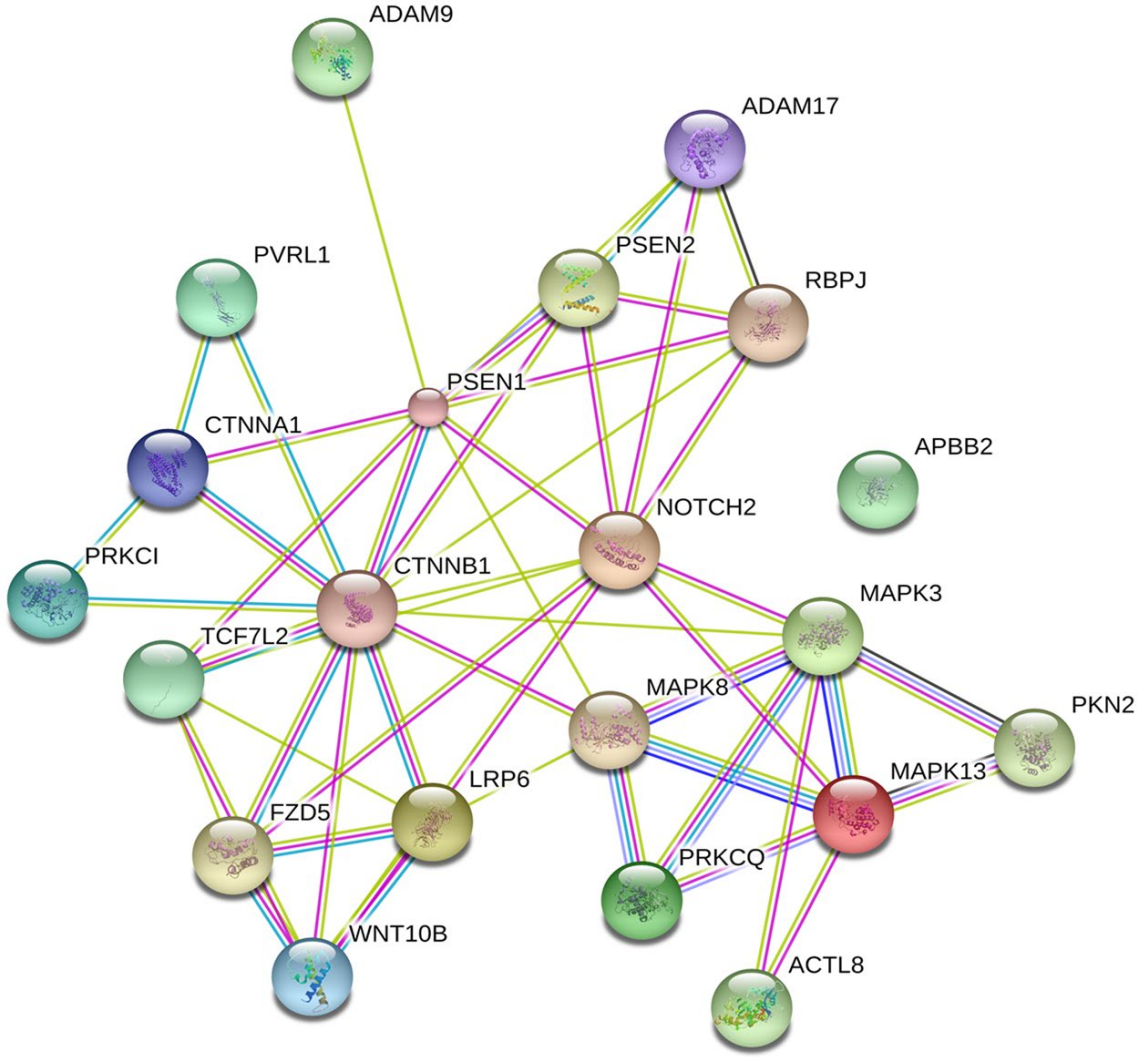
Network analysis of Alzheimer disease-amyloid secretase and of Alzheimer-disease presenilin genes catalogued in the Panther database and activated by Hp(2-20). Lines indicate interactions between proteins (or nodes).

*WNT10B* activates the canonical WNTs/β-catenin signaling pathway^34^, while *DKK1* - preventing LRP6 from interacting with WNTs^35^ – down-regulates the WNTs/β-catenin pathway. Attenuation of this pathway is known to favor the development of AD^36^. *TCF7L2* codes for a key transcription factor of the WNT signaling pathway^37^. *FZD5* is the receptor for the WNT5A ligand and participates in the β-catenin pathway induction^38,39^ (Fig. 7).

**Figure 8:**
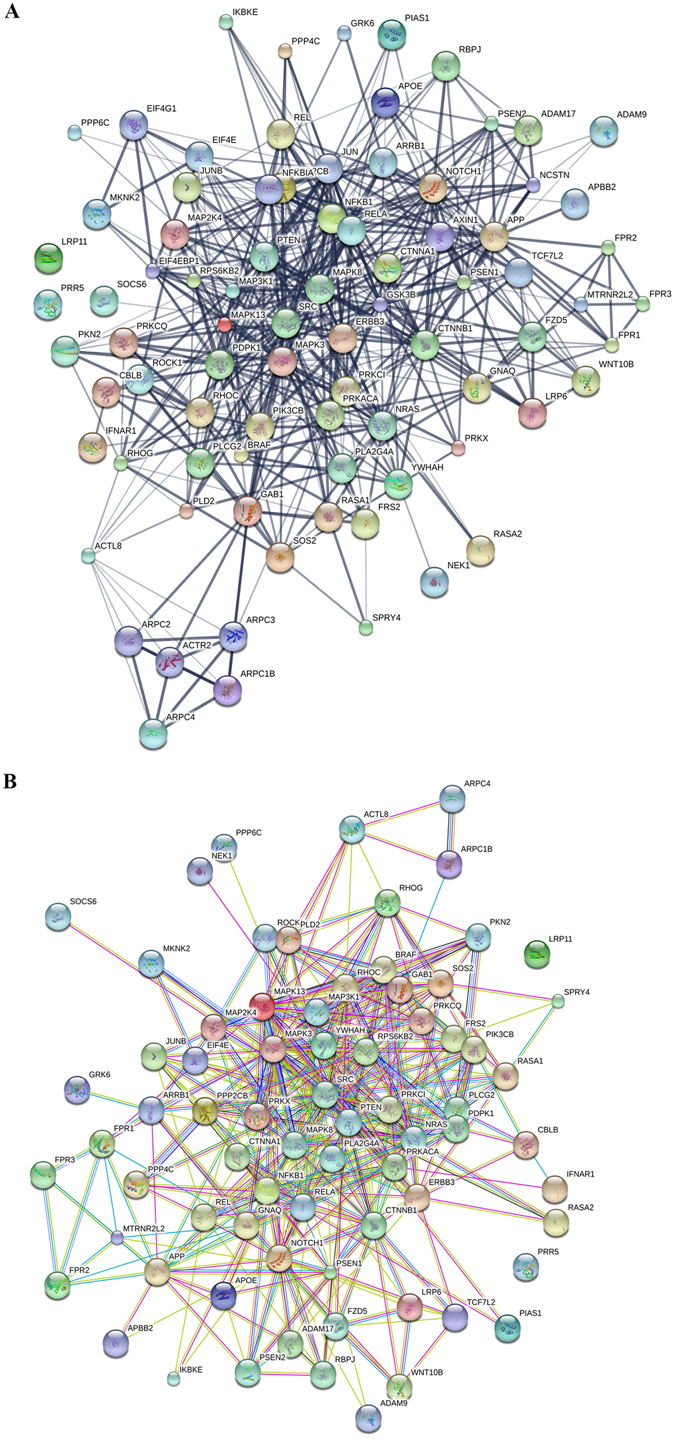
Network analysis of the gene pathways* catalogued in the Panther database, which - directly or indirectly - are connected with AD upon activation with Hp(2-20). Protein interactions were analyzed with the STITCH (**A**) and STRING (**B**) tools. The former catalogues known or predicted interactions between chemicals and proteins; the latter known or predicted protein-protein interactions. *List of the pathways included in the figure: Alzheimer disease-amyloid secretase pathway, Alzheimer disease-presenilin pathway, Angiogenesis, VEG-F signaling pathway, and Inflammation mediated by chemokine and cytokine signaling pathway.

*ANXA1* exerts a strong local anti inflammatory activity^40^. *APP, PSEN-1*, and *PSEN-2* mutations account for about 85% of EOAD cases^4^. The *APOE*-ε4 allele is a major risk factor for LOAD^5^. *CTNNB1* is present in several key biological pathways highly relevant to AD^41^. *MTRNR2L2* codes for the neuroprotective humanin protein, which in this study functions as a hub molecule for 17 molecules of the AD transcriptome (Fig. 8). FPR1 and FPRL1 are part of the AlzBase database and are both up-regulated in this study.

Finally, unique and total genes activated with Hp(2-20) - the most interesting category of genes – were studied by the ingenuity pathway analysis (IPA). We found that the unique – but not the total genes - target the amyloid processing pathway among the top five. This finding confirms that the peptide preferentially targets AD genes. Instead, total and unique genes do not display major differences with respect to the “top diseases and bio functions category”, suggesting that the peptide can induce *H. pylori* infection genes as well (Table 3).

**Table 3:** Pathways and functions activated by unique and total Hp(2-20) genes detected by IPA software.

Hp(2-20) peptide					
*Total*			*Unique*		
Top Canonical Pathways			Top Canonical Pathways		
Name	p-value	Overlap	Name	p-value	Overlap
EIF2 Signaling	5.05E-19	52.0% 115/221	Insulin Receptor Signaling	1.34E-06	22.0% 31/141
Regulation of eIF4 and p70S6K Signaling	1.22E-14	52.9% 83/157	Tight Junction Signaling	2.70E2.70E-06	20.4% 34/167
mTOR Signaling	6.16E-14	48.7% 97/199	Amyloid Processing	4.18E-06	31.40% 16/51
Protein Ubiquitination Pathway	2.82E-13	45.1% 115/255	IGF-1 Signaling	1.20E-05	22.6% 24/106
Glucocorticoid Receptor Signaling	3.35E-12	42.9% 123/287	RAR Activation	1.96E-05	18.4% 35/190
**Top Diseases and Bio Functions**			**Top Diseases and Bio Functions**		
***Diseases and Disorders***			***Diseases and Disorders***		
**Name**	**p-value**	**#Molecules**	**Name**	**p-value**	**#Molecules**
Cancer	1.50E-06-5.74E-101	4703	Cancer	7.67E-03-4.82E-28	1683
Organismal Injury and Abnormalities	1.67E-06-5.74E-101	4746	Organismal Injury and Abnormalities	7.67E-03-4.82E-28	1695
Gastrointestinal Disease	1.30E-06-1.75E-69	3977	Gastrointestinal Disease	7.67E-03-8.77E-17	1402
Infectious Diseases	1.93E-08-7.62E-31	796	Infectious Diseases	7.67E-03-1.08E-07	273
Developmental Disorder	8.60E-07-5.97E-23	644	Hematological Disease	7.67E-03-5.04E-05	151

### Modulation of the D-proline pathway genes

Microbial metabolites can reach the brain through direct interaction with enteric neurons^42^. D-proline, a metabolite of *H. pylori*^43^, ranks first among the 20 metabolites associated with AD^44^.

D-proline occurs at significantly higher levels (17.4 vs 2.4 nm/ml) in the gastric juice of patients with *H. pylori* infection compared to healthy controls^43^. While predisposing to AD^43,44^, D-proline improves the cognitive decline of AD patients^44^. These results open the possibility of using bacteria producing D-proline to curb the cognitive decline of AD patients. We found that both the Hp(2-20) peptide and *H. pylori* culture broth dis-regulate 14 and 12 of the genes participating to the synthesis of D-proline; besides, 70% of these genes are up-regulated (Supplementary Table S3). Thus, D-proline shows that the same metabolite can influence two different traits of the same disease in opposite directions; this unique trait of D-proline can permit to understand how this can occur.

### Genes dis-regulated in the Lesch-Nyhan and Alzheimer diseases

Several genes of the canonical WNT signaling, Alzheimer’s disease-presenilin, and Alzheimer disease-amyloid pathways are dis-regulated in both AD and Lesch-Nyhan disease^45–47^. Lesch-Nyhan is an incurable neurological disease caused by mutations of the hypoxanthine guanine phosphorybosyltransferase gene (*HPRT*)^45^. The canonical Wnt signaling pathway controls several aspects of vertebrate development (stem cell self-renewal, neurogenesis, and tumorigenesis)^48^ and has been associated with AD^49^. Dis-regulation of the Alzheimer’s disease-presenilin, and Alzheimer disease-amyloid pathways characterizes AD^3,4^. The former pathway also interferes with neural differentiation by stabilizing the β-catenin transcription^48^. Four of the 10 genes dis-regulated in Lesch-Nyhan disease and AD^45^ are also dis-regulated in the present study (Table 4). The lack of concordance in the transcriptional levels between the two studies very likely reflect differences in time-course.

**Table 4:** Genes dis-regulated in LND^1^ and AD. A comparison between two studies.

Gene Symbol	LND^2^	AD^3^	FDR	log2FC
*APOE*	Up	Up	2.56E-02	0.78
*ADAM9*	Up	Down	2.51E-02	−0.08
*LRP11*	Up	Up	4.31E-02	0.11
*PCLG2*	Up	Down	3.71E-02	−0.09
*CDK5R1*	Up	Nr^4^	Nr^4^	Nr^4^
*CAPN6*	Up	Nr	Nr^4^	Nr^4^
*ADAMTS4*	Up	Nr	Nr^4^	Nr^4^
*TNFRSF19*	Up	Nr	Nr^4^	Nr^4^
*BACE2*	Up	Nr	Nr^4^	Nr^4^
*PLCL2*	Down	Nr	Nr^4^	Nr^4^

^1^Leasch-Nyhan disease; ^2^Reference 64; ^3^This study; ^4^Nr = normo-regulated.

## Discussion

There is evidence – mainly inferred from apparently conflicting results between independent case-controls studies^13–15^ – that *H. pylori* infection might predispose to AD. To find a biologically plausible basis to this claim, we preconditioned the MNK-28 gastric cells with the Hp(2-20) peptide, the *H. pylori* culture broth or both and then interrogated the transcriptomes separately. To exclude possible artifacts deriving from interactions between bacterial components (please see the Results section), here we only discuss the data obtained with Hp (2-20) alone.

Why did we use the MNK-28 cells and Hp(2-20) peptide in the first place? First - to avoid confounding factors due to differences in immune response genes between hosts^50,51^ and the extreme differences existing between *H. pylori* strains^52^ - we opted for a less complex system: the transcriptome analysis of a human gastric cell line challenged with an *H. pylori* synthetic peptide. This experimental approach was also thought to facilitate replication of our results by independent workers. This when reproducibility is formally demanded in biomedical research^53^. Second, inflammation alters the permeability of the blood-brain barrier^54,55^ causing accumulation of the A$\beta$_42_ peptide in the microglia cells (the macrophages residing in the brain)^3,56^. A$\beta$_42_ is a regular resident in the brain where it exerts antimicrobial activity^57,58^, but – when in excess - stimulates inflammation and neuron apoptosis^59^. Thus, by activating the expression of the FPRL1 receptor on the MNK-28 cells with the Hp(2-20) ligand, we expected to probe two hallmarks of AD: inflammation and the Aβ_42_ plaque-forming process^26,60^.

How can *H. pylori* from the stomach affect the brain? There is credible evidence that the human microbiota communicates with the central nervous system through neural, endocrine, and immune pathways^61^. The proposed association of trimethylamine N-oxide (TMAO), succinic acid, mannitol^44^, and D-proline^43^ with AD is a demonstration that microbial metabolites might provoke AD. Also, it has been proposed that the immune response to *H. pylori* causes apoptosis and neural cell destruction by releasing pro-inflammatory molecules and inducing reactive oxygen metabolites^51,62^. There is evidence that *H. pylori* damages the brain-blood barrier^63,64^ and the gut metabolites reaches the brain through direct interaction with enteric neurons^42^. Microbial metabolites can also influence the peripheral immune response, which in sequence affects the blood- brain barrier^64^.

The peptide alone modulated 77 AD genes, of which 65 are listed in the AlzBase database (Table 5). This result excludes an experimental bias and proves the efficiency of the adopted RNA-seq approach. A large fraction of the modulated genes (30 out of 77) belong to the inflammation pathway (Table 1). This finding confirms what was anticipated above. Of the individual proteins that the 64 genes code for, here we discuss those deeply involved with AD and consequently more congruent with the objective of the present study.

**Table 5:** Profiles of 65 AD genes catalogued in the AlzBase database.

Gene	AlzBase				This study		Gene	AlzBase				This study	
	Sum	Up	Dn	Pe	FDR	Log2FC		Sum	Up	Dn	Pe	FDR	Log2FC
*ADAM17*	4	3	1	0	2.55E-02	−0.26	*PDPK1*	6	4	2	0	4.15E-02	−0.13
*ADAM9*	2	0	1	1	2.51E-02	−0.08	*PIAS1*	3	2	1	0	3.16E-02	−0.18
*ANXA1*	9	9	0	0	3.21E-02	0.13	*PIK3CB*	3	3	0	0	3.24E-02	−0.12
*APBB2*	3	0	3	0	2.99E-02	−0.18	*PKN2*	9	7	1	1	1.73E-02	−0.26
*APOE*	4	4	0	0	2.56E-02	0.78	*PLA2G4A*	2	2	0	0	3.12E-02	−0.17
*APP*	7	0	6	1	2.94E-02	0.04	*PLCG2*	4	4	0	0	4.27E-02	−0.2
*ARPC1B*	6	6	0	0	1.85E-02	0.18	*PLD2*	5	2	3	0	2.83E-02	0.15
*ARPC4*	3	0	3	0	2.86E-02	0.11	*PPP2CB*	4	0	4	0	3.79E-02	−0.08
*ARRB1*	9	5	3	1	2.29E-02	0.33	*PRKACA*	3	0	3	0	3.50E-02	0.14
*BRAF*	1	0	1	0	2.44E-02	−0.34	*PRKCI*	4	4	0	0	3.11E-02	−0.11
*CBLB*	8	6	1	1	3.19E-02	−0.29	*PRKCQ*	3	0	3	0	3.03E-02	−0.22
*CTNNA1*	6	4	2	0	1.87E-02	0.3	*PRKX*	10	9	0	1	3.11E-02	−0.2
*CTNNB1*	1	0	1	0	8.98E-03	−0.18	*PRR5*	2	1	1	0	2.88E-02	0.27
*EIF4E*	5	0	5	0	2.05E-02	−0.24	*PSEN1*	2	1	1	0	2.77E-02	0.35
*ERBB3*	7	6	1	0	4.24E-02	−0.08	*PSEN2*	6	0	4	2	1.83E-02	0.49
*FPR1*	8	6	2	0	3.06E-02	0.12	*PTEN*	1	1	0	0	3.26E-02	−0.11
*FPR2*	4	2	2	0	2.93E-02	0.59	*RASA1*	4	0	4	0	2.43E-02	−0.19
*FZD5*	3	3	0	0	4.99E-02	0.1	*RASA2*	5	1	4	0	3.11E-02	−0.28
*GAB1*	8	5	3	0	2.48E-02	−0.28	*RBPJ*	1	0	1	0	2.48E-02	−0.21
*GNAQ*	4	4	0	0	2.53E-02	−0.21	*REL*	2	1	1	0	2.42E-02	−0.54
*JUNB*	7	7	0	0	1.95E-02	0.34	*RELA*	8	7	0	1	4.35E-02	−0.09
*LRP11*	5	3	2	0	4.31E-02	0.11	*RHOC*	6	6	0	0	2.82E-02	0.21
*LRP6*	4	2	2	0	2.48E-02	−0.22	*RHOG*	7	7	0	0	4.91E-02	0.11
*MAP2K4*	11	1	10	0	2.48E-02	−0.26	*ROCK1*	5	5	0	0	3.11E-02	−0.14
*MAP3K1*	3	3	0	0	2.17E-02	0.23	*RPS6KB2*	2	2	0	0	2.84E-02	0.15
*MAPK13*	3	2	1	0	3.51E-02	0.1	*SOCS6*	2	0	2	0	4.28E-02	−0.16
*MAPK3*	2	0	2	0	1.52E-02	0.23	*SOS2*	3	3	0	0	2.41E-02	−0.29
*MAPK8*	9	2	6	1	4.24E-02	−0.15	*SPRY4*	6	1	5	0	2.85E-02	0.32
*MKNK2*	10	10	0	0	3.03E-02	0.13	*SRC*	3	2	1	0	2.39E-02	−0.18
*NEK1*	5	2	3	0	2.73E-02	−0.3	*TCF7L2*	3	3	0	0	3.14E-02	−0.18
*NFKB1*	9	8	0	1	2.35E-02	−0.15	*WNT10B*	1	1	0	0	2.72E-02	0.3
*NOTCH1*	14	14	0	0	1.21E-02	0.36	*YWHAH*	13	1	12	0	3.52E-02	0.08
*NRAS*	3	0	3	0	2.44E-02	−0.15							

Sum: Total number of differential expression from all transcriptome studies of Alzheimer’s disease (AD). Up: Total number of up regulation from all transcriptome studies of AD. Dn: Total number of down regulation from all transcriptome studies of AD. Pe: Total number of dys-regulation with unknown direction from all transcriptome studies of AD.

Because of its multiple roles in AD, we discuss first (and in more detail) ANXA1. This molecule is an endogenous ligand of the FPRL1 receptor^40^ but - at the transcriptional level - is directly connected with all three FPRs (Fig. 8). ANXA1 exerts a strong anti-inflammatory activity by promoting the removal of apoptotic neurons without inducing pro-inflammatory molecules (TNF-α, IL-6, and NO), a condition that limits local inflammation and spares healthy neurons^65,66^. ANXA1 accomplishes this task by bridging microglia cells through the FPRL1 receptor and apoptotic neurons by recognizing on their surface the phosphotidylserine, the “eat me” signal^40^. ANXA1 thus supports the protective role of phagocytosis (removal of apoptotic neurons) and at the same time curbs the negative effects of phagocytosis (loss of healthy neurons). Thus, the upregulation of ANXA1 by the Hp(2-20) peptide (observed in this study) and the ANXA1 accumulation in the brain of AD patients^40^ might be interpreted as compensatory mechanisms -occurring *in vitro* and *in vivo* - aimed to attenuate the side effects of inflammation. These results - along with concurrent ones^40^ - suggest a potential use of ANXA1 for the treatment of AD. In a mouse model of AD, the human recombinant ANXA1 (hrANXA1) has already displayed the property of repairing the blood–brain barrier integrity damaged by the Aβ_42_ peptide^67^.

MTRNR2L2 and ARRB1 are two more neuroprotective proteins. In this study, the former is down-regulated and the latter up-regulated. The humanin (MTRNR2L2) protein is directly connected to seven proteins and indirectly to ten more, all part of the AD transcriptome (Fig. 8). MTRNR2L2 is a 24 aminoacid polypeptide expressed in the occipital area of the brain; it recruits microglia cells to the site of inflammation to clear activated neutrophils^68^; induces Ca^++^ mobilization; and exerts neuroprotective activity, presumably by competing with the neurotoxic Aβ_42_ peptide for the FPRL1 receptor^68^. The down-regulation of *MTRNR2L2* observed in this study possibly reflects the characteristic of this gene to be highly expressed only in testis, kidney, skeletal muscles, and heart^69^. ARRB1 (β arrestin1) also displays neuroprotective activity during cerebral ischemic stress by regulating the BECN-dependent autophagosome formation^70^. In transgenic AD mice, ablation of the *ARRB1* gene reduces brain damage^71^.

Given the strict relationship between microbiome and neurodegenerative diseases including AD, we would like to explain why we did not attempt to identify *H. pylori* in the gut microbiome of AD patients. The gut microbiome generally is used to identify bacterial communities. The large difference in the number of *H. pylori* positive samples among healthy individuals reported in gastric microbiome^72^ and gut microbiome^73^ studies argues that faecal samples might be inadequate for *H. pylori* identification. Thus, a negative result of the gut microbiome assay would not necessarily exclude the presence of the pathogen. Further, it is well known that the human microbiome influences distant organs such as the brain through chemical signalling^44,74^. Excluded the use of biopsies for ethical reasons, we attempted to trace the link between H. pylori and AD indirectly, looking how the pathogen and its metabolites affect genes associated with AD.

AD and Lesch-Nyhan disease share several dis-regulated genes from the canonical Wnt signaling, Alzheimer-amyloid, and Alzheimer-presenilin pathways (Table 4). These results have been interpreted as evidence on a link between the two diseases. More recent data have shown that alternative splicing generates nine isoforms from the single copy *APP* gene^75^. PCR and sequencing techniques have demonstrated that the isoforms contain deletions that could affect the stability and function of the APP protein^75^. Given that APP is one of the proteins implicated in both AD and Lesch-Nyhan diseases, it has been speculated that the genetic diversity originated by the alternative splicing mechanism could potentially explain the clinical diversity and complexity of these diseases.

The link connecting *H. pylori* and AD emerged clearly only when the Hp(2-20) was used alone. In combination with the *H. pylori* growth broth, the genes hallmarks of AD (*APP, APOE, PSEN1*, *PSEN2, ANXA1, MTRNR2L2*) remained silent. If the Hp(2-20) silencing here observed occurs also *in vivo*, then the risk of AD attributable to *H. pylori* infection is expected to be one of the factors contributing to this multifactorial disease^76^.

In conclusion, here we identified 77 genes, 65 of which are listed in the AlzBase database. Remarkably, the pathways that result dis-regulated in AD and Leasch-Nyhan diseases in one study^68^ are dis-regulated in this one as well. The above data lend biological plausibility to the hypothesis of a connection between *H. pylori* infection and AD. The FPRL1 receptor and its ligand Aβ_42_ are part of this connection_._ *FPRL1* is expressed at high levels by the microglia cells infiltrating the brain tissue of AD patients^77^ and it has also been associated with AD^4^. The most difficult task will be to understand when inflammation and oxidant stress caused by the RPRL1- Aβ_42_ liaison is useful and when harmful to AD patients.

The unsuspected links between so different neurological diseases – though still awaiting formal validation – suggest new directions for these studies.

## Materials and Methods

### Bacteria and *H. pylori* growth broth

*H. pylori* strain ATCC 43504 was grown in 10 ml of liquid brain heart infusion medium (BHI; Oxoid, UK) supplemented with 10% foetal bovine serum (FBS; Oxoid), and incubated under microaerophilic condition generated by the CampyGen system (Oxoid) at 37 °C^78^. Bacteria were harvested at mid exponential phase, centrifuged (4.7 × 10^3^ g; 5 min), filtered (0.22 µ) and added (140 µl/well; 30 min and 1 h) to growing MKN-28 cells.

### Peptide

The cecropin-like peptide Hp(2-20) (sequence: NH2-AKKVFKRLEKLFSKIQNDK-COOH) corresponding to the amino-terminal part of the ribosomal protein L1 of *H. pylori* was synthesized by Innovagen (Lund, Sweden).

### Cell culture

The human gastric adenocarcinoma MKN-28 cell line, (ATCC, MD, USA) was grown in RPMI medium (Gibco, Scotland) supplemented with 10% FBS, penicillin (100 IU/ml) and streptomycin (100 μg/ml) (both from Gibco, Paisley, Scotland) at 37 °C in a 5% CO_2_ atmosphere. The cells were then distributed in a 24-well plate (10^5^ cells/well) (BD Falcon) and incubated (1 h) in the presence of the Hp(2-20) peptide (10^−5^ M)^25^, the *H. pylori* growth broth diluted 1:3 with serum-free medium, or both the peptide and the *H. pylori* growth broth.

### RNA extraction and Quantitative Real-time PCR

Total RNA was extracted from individual wells according to the TRIzol reagent protocol (Gibco/BRL Life Technologies, Inc., Gaithersburg, MD) and then reverse-transcribed using the high-Capacity cDNA Reverse transcription kit (Applied Biosystem). Expression levels of the FPR1 (Hs04235426_s1), FPRL1 (Hs02759175_s1), FPRL2 (Hs00266666_s1), and CTSG (Hs01113415_g1) were measured by rt-PCR using the TaqMan PCR master 2X reagent (Applied Biosystem) and the Applied Biosystem iCycler according to the manufacturer’s protocol. PCR reactions were carried out in triplicate. The TaqMan assay probes were from Life Technologies (Monza, Italy). Expression values were normalized versus the untreated MKN-28 (control) cells. The reference gene was the housekeeping GAPDH. Stability assay was carried out using the BestKeeper tool^79^.

### Transcriptome profiling with the RNA-Seq approach

Illumina reads were processed to remove adapter sequences and low quality bases (Phred score less than 25) by using Trimmomatic (version 0.33)^80^. The reads longer than 35 nt were retained for further analyses. Trimmed reads were then mapped against the human reference genome assembly (GRCh38.p3) from Ensembl version 82 with the program STAR (version 020201)^81^ The alignment files were filtered to retain the properly paired reads with a mapping quality higher than 30 by using SAMtools (version 1.2)^82^. Raw expression counts were then calculated by using featureCounts (version 1.4.6-p5)^83^. Raw counts were imported in R and, following TMM normalization, the lowly expressed genes were filtered out with the HTSFilter package^84^. Differential expression analysis of filtered genes was carried out with the NOISEQ package^85^. Gene Ontology Enrichment Analysis of differentially expressed genes was carried out using the human GO annotation from Ensembl version 82 and in-house-scripts. Significantly enriched GO categories were identified using the hyper geometric test.

## Electronic supplementary material

Supplementary Tables
