# Author Correction: The hypothesis that Helicobacter pylori predisposes to Alzheimer’s disease is biologically plausible

**DOI:** 10.1038/s41598-018-23613-x

**Published:** 2018-04-11

**Authors:** Felice Contaldi, Federico Capuano, Andrea Fulgione, Riccardo Aiese Cigliano, Walter Sanseverino, Domenico Iannelli, Chiara Medaglia, Rosanna Capparelli

**Affiliations:** 1 Department of Agriculture, University of Naples “Federico II”, Portici, 80055 Italy; 2 Department of Food Microbiology, Istituto Zooprofilattico Sperimentale del Mezzogiorno, Portici, 80055 Italy; 3 Sequentia Biotech, Edifici CRAG, Campus UAB, Bellaterra (Cerdanyola del Vallès), Barcelona, 08193 Spain; 4 Department of Immunology, Weizmann Institute of Science, Rehovot, 76100 Israel

Correction to: *Scientific Reports* 10.1038/s41598-017-07532-x, published online 10 August 2017

The original version of this Article contained errors in the spelling of the authors Felice Contaldi, Federico Capuano, Andrea Fulgione, Riccardo Aiese Cigliano, Walter Sanseverino, Domenico Iannelli, Chiara Medaglia & Rosanna Capparelli which were incorrectly given as Contaldi Felice, Capuano Federico, Fulgione Andrea, Aiese Cigliano Riccardo, Sanseverino Walter, Iannelli Domenico, Medaglia Chiara & Capparelli Rosanna.

These errors have now been corrected in the PDF and HTML versions of the Article, and in the accompanying Supplementary Material.

