# Supplementary material for: The hypothesis that Helicobacter pylori predisposes to Alzheimer’s disease is biologically plausible
Source: Sci Rep. 2017 Aug 10;7:7817. doi: 10.1038/s41598-017-07532-x (PMC5552707; doi:10.1038/s41598-017-07532-x)
Supplement: Supplementary file 1 — Supplementary Tables [file 41598_2017_7532_MOESM1_ESM.pdf]

Felice Contaldi[1, +], Federico Capuano[2, +], Andrea Fulgione[1], Riccardo Aiese Cigliano[3], Walter Sanseverino[3], Domenico Iannelli[1,*], Chiara Medaglia[4], Rosanna Capparelli[1,*]

[1]Department of Agriculture, University of Naples "Federico II", Portici, 80055, Italy

[2]Department of Food Microbiology, Istituto Zooprofilattico Sperimentale del Mezzogiorno, Portici, 80055, Italy.

[3] Sequentia Biotech, Edifici CRAG, Campus UAB, Bellaterra (Cerdanyola del Vallès), 08193, Spain.

[4]Department of Immunology, Weizmann Institute of Science, Rehovot, 76100, Israel.

[+] these authors contributed equally to this work

*corresponding authors:

Domenico Iannelli: iannelli@unina.it

Rosanna Capparelli: capparel@unina.it

Title: The hypothesis that *Helicobacter pylori* predisposes to Alzheimer's disease is biologically plausible.

Supplementary Table S1. Enriched GO terms associated with the 958 commons genes.

Process

GO term	Description	P-value	FDR q-value	Enrichment
GO:0006351	transcription, DNA-templated	0.0000914	0.524	1.53
GO:0097659	nucleic acid-templated transcription	0.0000914	0.262	1.53
GO:0045892	negative regulation of transcription, DNA-templated	0.000304	0.581	1.84
GO:0043408	regulation of MAPK cascade	0.000337	0.483	1.38
GO:0034616	response to laminar fluid shear stress	0.000595	0.682	7.98
GO:0034405	response to fluid shear stress	0.000595	0.568	7.98
GO:1904029	regulation of cyclin-dependent protein kinase activity	0.000645	0.528	23.71
GO:0000079	regulation of cyclin-dependent protein serine/threonine kinase activity	0.000645	0.462	23.71
GO:0072593	reactive oxygen species metabolic process	0.000686	0.437	89.56
GO:0006355	regulation of transcription, DNA-templated	0.000912	0.523	1.67
GO:1903507	negative regulation of nucleic acid-templated transcription	0.000984	0.512	1.76

Function

GO term	Description	P-value	FDR q-value	Enrichment
GO:0000977	RNA polymerase II regulatory region sequence-specific DNA binding	0.0000865	0.102	1.79
GO:0001012	RNA polymerase II regulatory region DNA binding	0.0000865	0.0512	1.79
GO:0001071	nucleic acid binding transcription factor activity	0.000348	0.137	1.53
GO:0003700	transcription factor activity, sequence-specific DNA binding	0.000348	0.103	1.53
GO:0000976	transcription regulatory region sequence-specific DNA binding	0.000396	0.0937	1.67
GO:0000981	RNA polymerase II transcription factor activity, sequence-specific DNA binding	0.000694	0.137	1.68
GO:1990837	sequence-specific double-stranded DNA binding	0.000728	0.123	1.64
GO:0043565	sequence-specific DNA binding	0.000753	0.111	1.58
GO:0043169	cation binding	0.000781	0.103	1.15
GO:0046872	metal ion binding	0.000781	0.0925	1.15
GO:0000987	core promoter proximal region sequence-specific DNA binding	0.000842	0.0906	1.88
GO:0001159	core promoter proximal region DNA binding	0.000842	0.083	1.88

Component

GO term	Description	P-value	FDR q-value	Enrichment
GO:0044425	membrane part	0.000117	0.0918	1.24
GO:0031224	intrinsic component of membrane	0.000517	0.203	1.28
GO:0016021	integral component of membrane	0.000681	0.179	1.28

*P--value' is the enrichment p-value computed according to the mHG or HG model.
**FDR q-value' is the correction of the above p-value for multiple testing using the Benjamini and Hochberg (1995) method. Namely, for the ith term (ranked according to p-value) the FDR q-value is (p-value * number of GO terms) / i.

Supplementary Table S2. Signaling pathways (131) represented among the 2066 dysregulated genes upon activation with Hp2-20.

Pathways	Gene number	PG*	PT**
SCW signaling pathway (P06216)	1	0.10%	0.10%
MYO signaling pathway (P06215)	1	0.10%	0.10%
GBB signaling pathway (P06214)	1	0.10%	0.10%
DPP signaling pathway (P06213)	1	0.10%	0.10%
DPP-SCW signaling pathway (P06212)	1	0.10%	0.10%
BMP/activin signaling pathway-drosophila (P06211)	1	0.10%	0.10%
Activin beta signaling pathway (P06210)	1	0.10%	0.10%
Axon guidance mediated by netrin (P00009)	3	0.20%	0.40%
Axon guidance mediated by Slit/Robo (P00008)	3	0.20%	0.40%
Axon guidance mediated by semaphorins (P00007)	3	0.20%	0.40%
Apoptosis signaling pathway (P00006)	23	1.20%	3.00%
Pyridoxal-5-phosphate biosynthesis (P02759)	1	0.10%	0.10%
Gonadotropin-releasing hormone receptor pathway (P06664)	32	1.70%	4.20%
Angiogenesis (P00005)	25	1.30%	3.30%
Ornithine degradation (P02758)	2	0.10%	0.30%
Alzheimer disease-presenilin pathway (P00004)	14	0.70%	1.80%
Alzheimer disease-amyloid secretase pathway (P00003)	10	0.50%	1.30%
Alpha adrenergic receptor signaling pathway (P00002)	1	0.10%	0.10%
Methylmalonyl pathway (P02755)	1	0.10%	0.10%
Adrenaline and noradrenaline biosynthesis (P00001)	2	0.10%	0.30%

Methionine biosynthesis (P02753)	1	0.10%	0.10%
Mannose metabolism (P02752)	2	0.10%	0.30%
CCKR signaling map (P06959)	24	1.20%	3.20%
Ubiquitin proteasome pathway (P00060)	11	0.60%	1.40%
ALP23B signaling pathway (P06209)	1	0.10%	0.10%
Leucine biosynthesis (P02749)	1	0.10%	0.10%
p53 pathway (P00059)	14	0.70%	1.80%
Isoleucine biosynthesis (P02748)	1	0.10%	0.10%
mRNA splicing (P00058)	2	0.10%	0.30%
Wnt signaling pathway (P00057)	33	1.70%	4.30%
Heme biosynthesis (P02746)	3	0.20%	0.40%
VEGF signaling pathway (P00056)	9	0.50%	1.20%
Glutamine glutamate conversion (P02745)	1	0.10%	0.10%
Transcription regulation by bZIP transcription factor (P00055)	9	0.50%	1.20%
Fructose galactose metabolism (P02744)	4	0.20%	0.50%
Toll receptor signaling pathway (P00054)	9	0.50%	1.20%
Formyltetrahydroformate biosynthesis (P02743)	1	0.10%	0.10%
T cell activation (P00053)	9	0.50%	1.20%
TGF-beta signaling pathway (P00052)	17	0.90%	2.20%
TCA cycle (P00051)	1	0.10%	0.10%
De novo pyrimidine ribonucleotides biosythesis (P02740)	1	0.10%	0.10%
Plasminogen activating cascade (P00050)	1	0.10%	0.10%
De novo pyrimidine deoxyribonucleotide biosynthesis (P02739)	2	0.10%	0.30%

Parkinson disease (P00049)	12	0.60%	1.60%
De novo purine biosynthesis (P02738)	4	0.20%	0.50%
PI3 kinase pathway (P00048)	7	0.40%	0.90%
PDGF signaling pathway (P00047)	27	1.40%	3.50%
Coenzyme A biosynthesis (P02736)	3	0.20%	0.40%
Oxidative stress response (P00046)	10	0.50%	1.30%
Notch signaling pathway (P00045)	6	0.30%	0.80%
Nicotinic acetylcholine receptor signaling pathway (P00044)	7	0.40%	0.90%
Muscarinic acetylcholine receptor 2 and 4 signaling pathway (P00043)	7	0.40%	0.90%
Muscarinic acetylcholine receptor 1 and 3 signaling pathway (P00042)	5	0.30%	0.70%
Metabotropic glutamate receptor group I pathway (P00041)	6	0.30%	0.80%
Asparagine and aspartate biosynthesis (P02730)	1	0.10%	0.10%
Metabotropic glutamate receptor group II pathway (P00040)	5	0.30%	0.70%
Synaptic vesicle trafficking (P05734)	4	0.20%	0.50%
GABA-B receptor II signaling (P05731)	5	0.30%	0.70%
Ascorbate degradation (P02729)	1	0.10%	0.10%
Metabotropic glutamate receptor group III pathway (P00039)	6	0.30%	0.80%
JAK/STAT signaling pathway (P00038)	1	0.10%	0.10%
Ionotropic glutamate receptor pathway (P00037)	3	0.20%	0.40%
Interleukin signaling pathway (P00036)	12	0.60%	1.60%
Interferon-gamma signaling pathway (P00035)	7	0.40%	0.90%
Alanine biosynthesis (P02724)	1	0.10%	0.10%
Xanthine and guanine salvage pathway (P02788)	1	0.10%	0.10%

Adenine and hypoxanthine salvage pathway (P02723)	2	0.10%	0.30%
Integrin signalling pathway (P00034)	21	1.10%	2.80%
Vitamin B6 metabolism (P02787)	1	0.10%	0.10%
Acetate utilization (P02722)	1	0.10%	0.10%
Insulin/IGF pathway-protein kinase B signaling cascade (P00033)	3	0.20%	0.40%
Insulin/IGF pathway-mitogen activated protein kinase kinase/MAP kinase cascade (P00032)	6	0.30%	0.80%
p53 pathway feedback loops 2 (P04398)	9	0.50%	1.20%
Valine biosynthesis (P02785)	1	0.10%	0.10%
Inflammation mediated by chemokine and cytokine signaling pathway (P00031)	27	1.40%	3.50%
p53 pathway by glucose deprivation (P04397)	4	0.20%	0.50%
Hypoxia response via HIF activation (P00030)	3	0.20%	0.40%
Vitamin D metabolism and pathway (P04396)	2	0.10%	0.30%
Vasopressin synthesis (P04395)	1	0.10%	0.10%
Thyrotropin-releasing hormone receptor signaling pathway (P04394)	5	0.30%	0.70%
Thiamin metabolism (P02780)	1	0.10%	0.10%
Ras Pathway (P04393)	13	0.70%	1.70%
P53 pathway feedback loops 1 (P04392)	1	0.10%	0.10%
Oxytocin receptor mediated signaling pathway (P04391)	5	0.30%	0.70%
Huntington disease (P00029)	16	0.80%	2.10%
Heterotrimeric G-protein signaling pathway-rod outer segment phototransduction (P00028)	2	0.10%	0.30%
Heterotrimeric G-protein signaling pathway-Gq alpha and Go alpha mediated pathway (P00027)	10	0.50%	1.30%
p38 MAPK pathway (P05918)	4	0.20%	0.50%

Heterotrimeric G-protein signaling pathway-Gi alpha and Gs alpha mediated pathway (P00026)	12	0.60%	1.60%
Opioid proopiomelanocortin pathway (P05917)	1	0.10%	0.10%
Hedgehog signaling pathway (P00025)	3	0.20%	0.40%
Sulfate assimilation (P02778)	1	0.10%	0.10%
Opioid prodynorphin pathway (P05916)	1	0.10%	0.10%
Glycolysis (P00024)	3	0.20%	0.40%
Succinate to proprionate conversion (P02777)	1	0.10%	0.10%
Opioid proenkephalin pathway (P05915)	1	0.10%	0.10%
General transcription regulation (P00023)	6	0.30%	0.80%
Nicotine pharmacodynamics pathway (P06587)	4	0.20%	0.50%
General transcription by RNA polymerase I (P00022)	3	0.20%	0.40%
Salvage pyrimidine ribonucleotides (P02775)	2	0.10%	0.30%
Enkephalin release (P05913)	2	0.10%	0.30%
FGF signaling pathway (P00021)	24	1.20%	3.20%
Salvage pyrimidine deoxyribonucleotides (P02774)	2	0.10%	0.30%
Dopamine receptor mediated signaling pathway (P05912)	6	0.30%	0.80%
FAS signaling pathway (P00020)	5	0.30%	0.70%
Angiotensin II-stimulated signaling through G proteins and beta-arrestin (P05911)	5	0.30%	0.70%
Histamine H2 receptor mediated signaling pathway (P04386)	2	0.10%	0.30%
Histamine H1 receptor mediated signaling pathway (P04385)	4	0.20%	0.50%
Pyrimidine Metabolism (P02771)	2	0.10%	0.30%
Gamma-aminobutyric acid synthesis (P04384)	1	0.10%	0.10%

Pyridoxal phosphate salvage pathway (P02770)	1	0.10%	0.10%
Cortocotropin releasing factor receptor signaling pathway (P04380)	2	0.10%	0.30%
Endothelin signaling pathway (P00019)	11	0.60%	1.40%
EGF receptor signaling pathway (P00018)	25	1.30%	3.30%
DNA replication (P00017)	4	0.20%	0.50%
Cytoskeletal regulation by Rho GTPase (P00016)	9	0.50%	1.20%
Circadian clock system (P00015)	1	0.10%	0.10%
Cholesterol biosynthesis (P00014)	3	0.20%	0.40%
Cell cycle (P00013)	4	0.20%	0.50%
Cadherin signaling pathway (P00012)	13	0.70%	1.70%
Beta3 adrenergic receptor signaling pathway (P04379)	1	0.10%	0.10%
Blood coagulation (P00011)	2	0.10%	0.30%
Beta2 adrenergic receptor signaling pathway (P04378)	3	0.20%	0.40%
B cell activation (P00010)	11	0.60%	1.40%
Beta1 adrenergic receptor signaling pathway (P04377)	3	0.20%	0.40%
5HT4 type receptor mediated signaling pathway (P04376)	1	0.10%	0.10%
Pentose phosphate pathway (P02762)	2	0.10%	0.30%
5HT3 type receptor mediated signaling pathway (P04375)	1	0.10%	0.10%
5HT2 type receptor mediated signaling pathway (P04374)	5	0.30%	0.70%
5HT1 type receptor mediated signaling pathway (P04373)	3	0.20%	0.40%
5-Hydroxytryptamine degredation (P04372)	2	0.10%	0.30%

*Percent of gene hit against total genes.

**Percent of gene hit against total pathway hits.
